# National Trends in the Use of State-Reimbursed Lipid-Lowering Medications in Latvia (2012–2021)

**DOI:** 10.3390/jcm12196390

**Published:** 2023-10-06

**Authors:** Arturs Praskilevics, Inga Urtane, Gustavs Latkovskis

**Affiliations:** 1 Department of Pharmaceutical Chemistry, Faculty of Pharmacy, Riga Stradins University, LV-1007 Riga, Latvia; inga.urtane@rsu.lv; 2 Red Cross Medical College, Riga Stradins University, LV-1009 Riga, Latvia; 3 Institute of Cardiology and Regenerative Medicine, University of Latvia, LV-1004 Riga, Latvia; gustavs.latkovskis@gmail.com; 4 Latvian Center of Cardiology, Pauls Stradins Clinical University Hospital, LV-1002 Riga, Latvia; 5 Faculty of Medicine, University of Latvia, LV-1004 Riga, Latvia

**Keywords:** lipid-lowering medications, trends, dyslipidemia, statins, ezetimibe, fixed-dose combination, PCSK9 inhibitors, fenofibrate

## Abstract

Background. We aimed to estimate the trends in dispensing rate and the spectrum of all state-funded lipid-lowering medications (LLMs) in Latvia over a decade. Methods. Using data from the National Health Service of the Republic of Latvia, we retrospectively analyzed all dispensed LLM-containing drug units in a ten-year period from 2012 to 2021. Results. In Latvia, 318.2 million oral and 994 subcutaneous units of LLMs were dispensed over a decade. Statins were the most dispensed LLMs (94.5%), and their use doubled from 19.7 to 43.5 million units. The proportion of high-intensity statins increased from 31.3% to 45.2%. The dispensing rate of ezetimibe increased from 184.7 thousand to 4.8 million. The share of fixed-dose statin combinations with ezetimibe grew from 0.2% to 10.0% among all statins and from 22.2% to 90.9% among all ezetimibe units. Statin use for primary and secondary prevention increased from 7.0 to 19.9 million and from 12.8 to 23.6 million units, respectively. Conclusion. The dispensing rate of statins doubled, and the use of ezetimibe increased more than 25 times in Latvia over a decade. The proportion of high-intensity statins increased from one third to almost half of all statins. Fixed-dose statin combinations with ezetimibe became frequently used.

## 1. Introduction

Cardiovascular diseases (CVD) cause around 39% of all deaths in men and 46% of all deaths in women in Europe [1]. The global consumption of lipid-lowering medications (LLM) continues increase to reduce lipid levels and prevent the risk of atherosclerotic cardiovascular disease (ASCVD). The European Society of Cardiology and the European Atherosclerosis Society 2019 guidelines for the treatment of dyslipidemia indicate statins as first-line drugs for primary and secondary prevention of ASCVD; however, in order to achieve the contemporary low-density lipoprotein-cholesterol (LDL-cholesterol) goals it is often necessary to use combinations of LLM [2]. Both European and American International guidelines recommend lower LDL-cholesterol targets and a more frequent need for high-intensity statin therapy and combinations [2,3,4]. Intensive LDL-cholesterol lowering is particularly relevant in secondary prevention.

Monitoring and analyzing drug consumption data at the national level allows us to assess the dynamics of drug consumption in the country as well as compliance with international guidelines. Moreover, knowledge of these trends assists in planning the state budget for reimbursed pharmaceuticals and their rational usage. However, the data on true volumes and consumption of LLMs in most countries of the world are scarce [5]. A cross-sectional and ecological study that collected information on global trends and consumption of lipid-lowering drugs in 83 countries found that between 2008 and 2018, the consumption of LLMs increased from 7468 to 11,197 standard units per 1000 individuals per year. It was estimated that in 2018, 173 million inhabitants of the analyzed countries used at least one LLM [5]. There are few similar studies, and there is a substantial knowledge gap in trends of LLM use in the Baltic region and the North-East of Europe.

To our knowledge, no previous study has addressed the consumption of LLMs in Latvia. Here, we aimed to estimate trends in the dispensing rate and spectrum of all state-reimbursed LLMs in Latvia from 2012 to 2021. The objectives were to calculate amounts and trends of all dispensed LLMs and their doses for ten years, to analyze the prevalence of high-intensity statins and fixed-dose combinations among all LLMs, and to estimate LLMs dispensed for primary and secondary prevention.

## 2. Materials and Methods

Publicly available databases of all state-reimbursed prescriptions dispensed in Latvia from 1 January 2012 to 31 December 2021, were obtained from the National Health Service of the Republic of Latvia [6]. In a retrospective analysis, data on all LLM-containing drugs were extracted, including fixed-dose combinations of a statin with ezetimibe and a statin with one or more antihypertensive agents. No specific inclusion or exclusion criteria were applied. The following data were available in the database: international nonproprietary name (INN), trade name, dose, manufacturer, the applicant of registration, ICD-10 (International Classification of Diseases 10th Revision) code of the diagnosis it was dispensed for, degree of reimbursement, package identification number, the annual number of dispensed packages, annual number of recipes and the total amount covered by the state in euros. For data analysis, the term “unit” was used, which was a single tablet, capsule, or injection vial. The number of units per package was obtained based on the package identification number. The totality of units was calculated by multiplying the number of dispensed packages and the number of units per package. 

Atorvastatin 40–80 mg or rosuvastatin 20–40 mg was defined as high-intensity statin therapy, and other doses we considered non-high-intensity statins according to the guidelines of the American College of Cardiology (ACC) and the American Heart Association (AHA) [7]. The definitions of primary and secondary prevention based on ICD-10 classification codes are given in Table S1. For example, LLM prescribed for the diagnosis code E78.0 (pure hypercholesterolaemia) was considered as the primary prevention case, and a prescription for I21 (acute myocardial infarction) was the secondary prevention case. An example of non-classifiable cases that were attributed to neither primary, nor secondary prevention is Z95.0 (presence of electronic cardiac devices). 

There was no individual patient participation in the design. Approval of the ethics committee was not relevant as only publicly available aggregate data were used and no sensitive patient data were obtained or exposed. 

The data were analyzed using IBM SPSS Statistics for Windows, version 29 (IBM Corp., Armonk, NY, USA). In descriptive analysis, categorical variables were expressed as counts and percentages. Continuous variables were expressed as means and standard deviations. Changes in continuous variables were compared with the paired samples *t*-test. *p* values below 0.05 were considered statistically significant.

## 3. Results

### 3.1. Dispensing Rates of LLMs and the Spectrum of Doses

In total, 318.2 million units of oral LLMs (statins, ezetimibe, and fenofibrate) were dispensed in Latvia from 2012 to 2021, and 994 units of PCSK9 inhibitors (alirocumab and evolocumab) over a 2-year period from 2020 to 2021. The majority of all dispensed LLMs were statins: 300.7 million units (94.5%). The detailed numbers of units per dose and year are summarized in Table S2.

**Figure 1:**
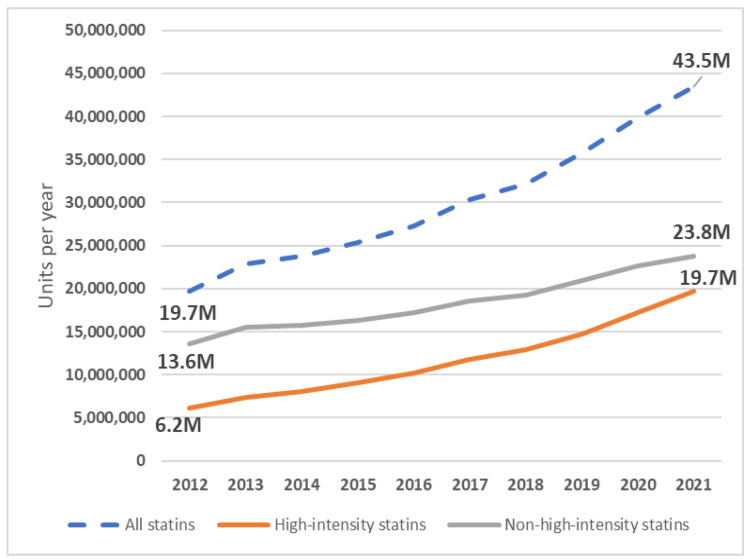
Annual dispensing rate of high- and non-high-intensity statins over a 10-year period. M—million.

**Figure 2:**
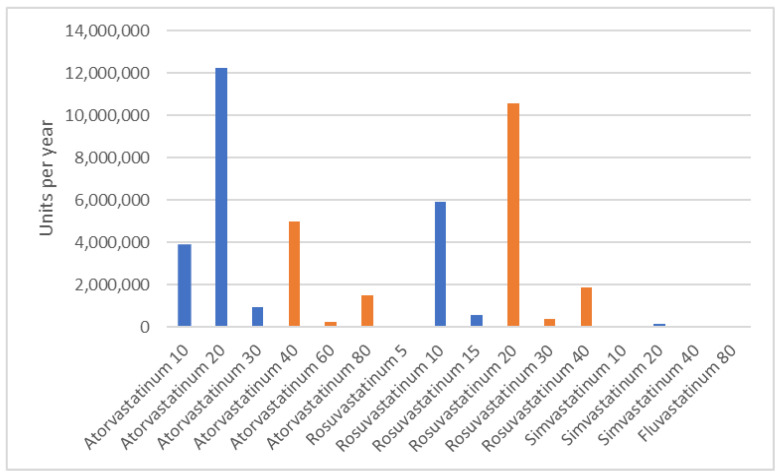
Most dispensed statins in 2021. Orange bars—high-intensity statins, blue bars—non-high-intensity statins.

Over a decade, the absolute number of statins doubled from 19.7 to 43.5 million units per year (Figure 1). Among satins, the share of atorvastatin and rosuvastatin increased from 97.0% to 99.6%. The proportion of atorvastatin decreased from 79.6% to 54.9% and the proportion of rosuvastatin increased from 17.4% to 44.7% in ten years. The total amount of dispensed atorvastatin increased from 15.7 million units in 2012 to 23.9 million units in 2021. Rosuvastatin usage increased more rapidly: from 3.4 million units in 2012 to 19.4 million units in 2021. The most commonly dispensed statins in 2021 were atorvastatin 20 mg, rosuvastatin 20 mg, and rosuvastatin 10 mg (12.3 million, 10.6 million, and 5.9 million units, respectively, Figure 2), as opposed to atorvastatin 20 mg, atorvastatin10 mg, and atorvastatin 40 mg in 2012 (7.4 million, 4.1 million and 3.4 million units, respectively). Simvastatin use declined from 554.9 thousand units in 2012 to 181.2 thousand units in 2021. Fluvastatin dispensation also continued to decrease from 38.3 thousand units in 2012 to 9.8 thousand units in 2021. No other statins were reimbursed during this time period.

**Figure 3:**
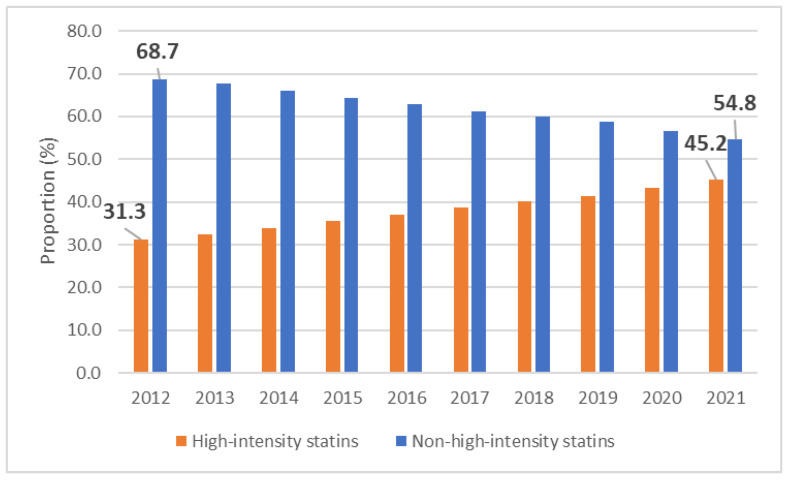
Proportion of high and non-high-intensity statin dispensation in a decade. Orange bars—high-intensity statins, blue bars—non-high-intensity statins.

The dispensing rate of high-intensity statins increased from 6.2 million in 2012 to 19.7 million in 2021 (Figure 1). The corresponding share of high-intensity statins increased from 31.3% in 2012 to 45.2% in 2021 (Figure 3). However, an increase was also observed in the consumption of non-high-intensity statins: in 2021 it almost doubled to 23.8 million units in 2021 compared with 13.6 million units in 2012 (Figure 1). When analyzing maximal doses, over the 10-year period, the dispensing rate of atorvastatin 80 mg increased from 723.3 thousand units in 2012 to 1.5 million units in 2021. The respective quantity of rosuvastatin 40 mg increased from 226.7 thousand units in 2012 to 1.9 million units in 2021. The highest rate for atorvastatin 80 mg was 1.8 million units in 2018 and for rosuvastatin 40 mg it was 1.9 million units in 2021. 

Atorvastatin 80 mg constituted 4.6% in 2012 and 6.4% in 2021 among all dispensed atorvastatins, with a peak of 8.1% in 2018. Rosuvastatin 40 mg constituted 6.6% in 2012 and 9.6% in 2021 among all dispensed rosuvastatins, with a peak of 13.6% in 2018. The mean weighted dose in 2012 was 24.6 ± 16.1 mg for atorvastatin and 17.5 ± 7.8 mg for rosuvastatin, while in 2021 it was 27.2 ± 17.3 mg and 18.9 ± 8.4 mg, respectively (*p* < 0.001 for both). The weighted doses of both statins peaked in 2018: 28.1 ± 18.6 mg for atorvastatin and 19.7 ± 9.4 mg for rosuvastatin.

**Figure 4:**
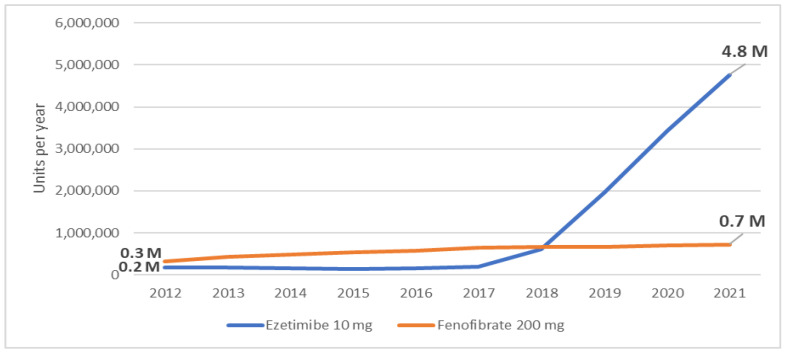
Dispensing rate of ezetimibe and fenofibrate in the ten-year period. M—million.

The dispensing rate of ezetimibe 10 mg increased by a factor of 25.8 from 184.7 thousand units in 2012 to 4.8 million units in 2021 (Figure 4). The use of fenofibrate 200 mg had more than doubled but remained low compared to statins and ezetimibe: 722.1 thousand units in 2021 compared to 319.2 thousand units in 2012 (Figure 4). Alirocumab and evolocumab, two PCSK9 inhibitors, have been reimbursed in Latvia since July 2020. In 2020, 10 and 38 units of 75 mg and 150 mg Alirocumab were dispensed, respectively. In 2021, these numbers of units were 76 and 392, respectively. The corresponding unit numbers for evolocumab 140 mg were 114 in 2020 and 364 in 2021. No other non-statin LLMs (such as inclisiran or bempedoic acid) were reimbursed in the studied period. 

### 3.2. Fixed-Dose Combinations of LLMs

**Figure 5:**
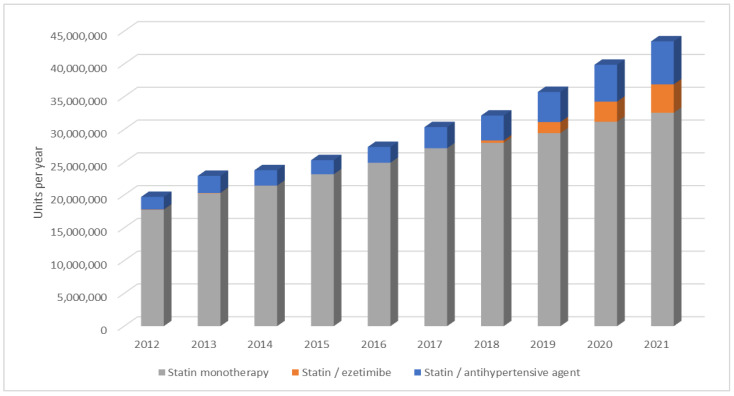
Dispensation of statins in monotherapy and in fixed-dose combinations (2012–2021).

Annual statin dispensing rate had grown in both monotherapy and fixed-dose combination (FDC) groups (Figure 5). In a decade, statin FDCs with ezetimibe grew from 41.0 thousand in 2012 to 4.3 million in 2021. In total, 9.6 million statin units were dispensed in FDCs with ezetimibe over a decade. In Latvia, FDCs with ezetimibe were available for simvastatin from 2012 to 2014. There were no FDCs with ezetimibe available in the period of 2015–2017. Fixed-dose combinations became available with rosuvastatin in 2018 and with atorvastatin in 2020. Simvastatin FDCs with ezetimibe were no longer available in Latvia from 2015. The fraction of LLMs that were 100% compensated gradually decreased from 15.6% in 2012 to 15.2% in 2020 and 14.6% in 2021.

Since FDCs became available with rosuvastatin in 2018 and atorvastatin in 2020, a continuous increase has been observed in dispensation of both high-intensity (from 244.0 thousand in 2018 to 3.0 million units in 2021) and non-high-intensity statin combinations (increased from 1193 thousand in 2018 to 1.4 million units in 2021). The proportion of high-intensity statins among all statin/ezetimibe combinations increased modestly from 67.2% in 2018 to 68.3% in 2021.

**Figure 6:**
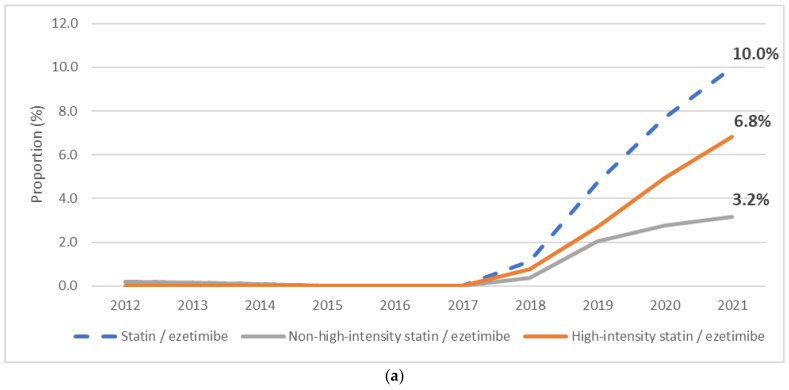

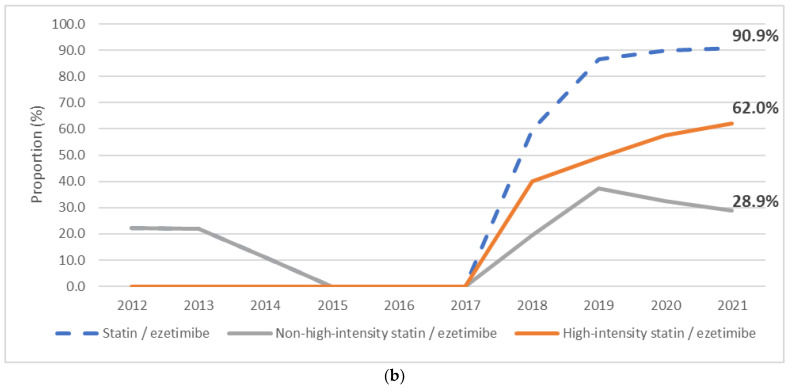
The proportion of fixed-dose combinations of statin/ezetimibe among all statins (**a**) and all ezetimibes (**b**) in the period of 2012–2021.

Among all dispensed statins, the proportion of statin FDCs with ezetimibe grew from 0.2% in 2012 to 10.0% in 2021: 6.8% with high-intensity statins and 3.2% with non-high intensity statins (Figure 6a). Of all dispensed ezetimibe units, the proportion of FDCs with statins quadrupled from 22.2% in 2012 to 90.9% in 2021: 62.0% with high-intensity statins and 28.9% with non-high intensity statins (Figure 6b). After becoming commercially available in 2018, the share of high-intensity statin FDCs with ezetimibe among all statin/ezetimibe combinations increased from 40.0% to 62.0% by 2021. 

### 3.3. Fixed-Dose Combinations of Statins with Antihypertensive Drugs

In total, 35.2 million units of statin FDCs with antihypertensives were dispensed from 2012 to 2021. Only atorvastatin and rosuvastatin were available as statin FDCs with antihypertensive agents during the reported period: atorvastatin/amlodipine, atorvastatin/perindopril, atorvastatin/perindopril/amlodipine, rosuvastatin/amlodipine, rosuvastatin/amlodipine/perindopril and rosuvastatin/perindopril/indapamide. The total number of statin/antihypertensive agent FDCs increased from 1.9 million in 2012 to 6.6 million in 2021. Before 2016, only atorvastatin was available in combination with antihypertensive agents. Since 2016, rosuvastatin in combination with antihypertensive agents has also been marketed in Latvia. Rosuvastatin has not been available in Latvia in combinations with one antihypertensive drug since 2020. 

**Figure 7:**
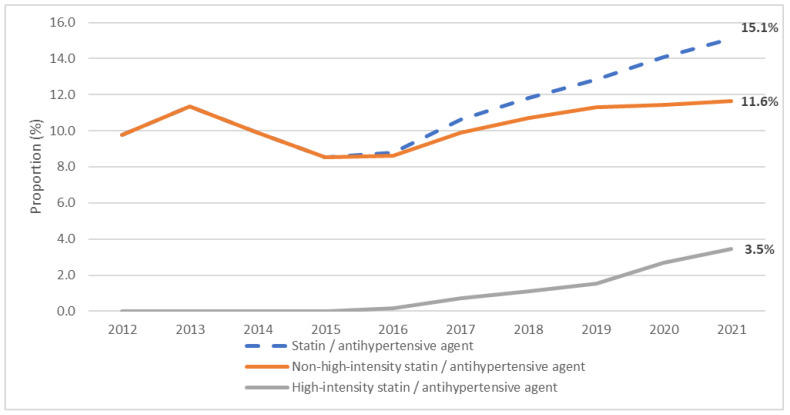
Proportion of statin/antihypertensive agent fixed-dose combinations among all statins (2012–2021).

**Figure 8:**
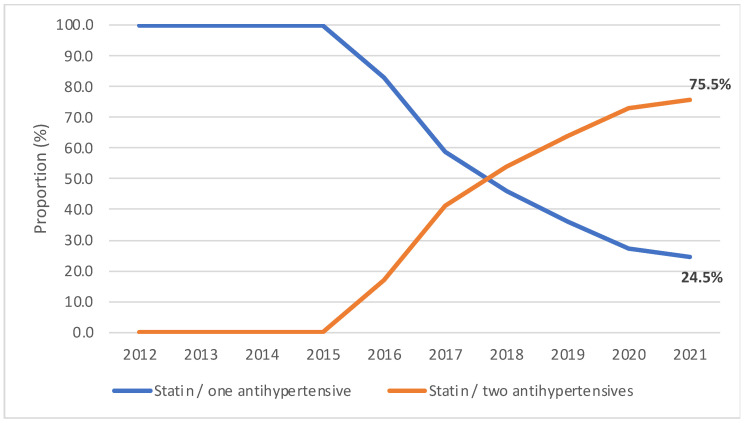
Proportions of statin combinations with one and two antihypertensive agents among all combinations of statin/antihypertensive agent (2012–2021).

The proportion of statin FDCs with one or more antihypertensive agents out of all dispensed statins continued increasing from 9.8% in 2012 to 15.1% in 2021 (Figure 7). Among all statins, the proportion of statin combinations with one antihypertensive drug decreased threefold from 9.8% in 2012 to 3.7% in 2021. Statin combinations with 2 antihypertensive drugs became available in 2016 and the share among all statins increased from 1.5%% to 11.4% in 2021. Statin FDCs with two antihypertensive agents constituted 17.1% of all statin/antihypertensive agent combinations in 2016, but this proportion increased to 75.5% in 2021 (Figure 8). 

High-intensity statins in combination with antihypertensive drugs were dispensed since 2016. The dispensation of high-intensity statins in combination with antihypertensive drugs increased almost 30 times from 2016 to 2021: from 50.7 thousand units in 2016 to 1.5 million units in 2021. However, the quantity of non-high-intensity statin combinations with antihypertensive drugs had nearly tripled between 2012 and 2021 (from 1.9 million units in 2012 and 5.1 million units in 2021). 

### 3.4. Primary and Secondary CVD Prevention

**Figure 9:**
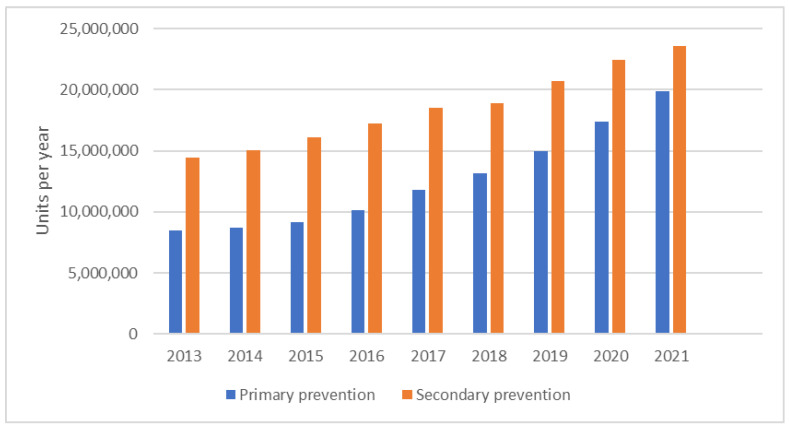
Annual dispensing rate of statins according to the type of cardiovascular prevention from 2012 to 2021.

From 2012 to 2021, the total dispensation of all statins tripled from 7.0 million units to 19.9 million for primary prevention and doubled from 12.8 million units to 23.6 million for secondary prevention (Figure 9). Ezetimibe became reimbursed for primary CVD prevention in 2020 only for patients with familial hypercholesterolemia, and the number of dispensed units increased from 5.9 to 16.4 thousand in two years. The amount of ezetimibe dispensed for secondary CVD prevention rose from 184.7 thousand units in 2012 to 4.8 million units in 2021. 

The proportion of high-intensity statins for the primary prevention of CVD was 17.0% of all statins in primary prevention in 2012, and it doubled to 34.4% in 2021. The proportion of high-intensity statins for secondary CVD prevention increased from 39.0% in 2012 to 54.3% in 2021. In 2012, the mean weighted dose in primary prevention was 18.0 ± 10.7 mg for atorvastatin and 16.4 ± 7.2 mg for rosuvastatin, while in 2021 it was 22.3 ± 11.9 mg and 18.2 ± 8.0 mg, respectively (*p* < 0.001 for both). In secondary prevention, from year 2012 to 2021, the mean weighted dose of atorvastatin and rosuvastatin increased from 28.4 ± 17.4 mg to 32.4 ± 20.3 mg and from 18.0 ± 7.9 mg to 19.4 ± 8.7 mg, respectively (*p* < 0.001 for both). 

Additional analysis was performed to calculate dispensing habits of maximal doses of statins among all respective statin doses in prevention subgroups. 

The absolute amounts of dispensed atorvastatin 80 mg continued increasing during the whole decade for primary prevention, but in secondary prevention they peaked in 2018 and then declined to the levels before year 2017 (Figure S1). The absolute amounts of dispensed rosuvastatin 40 mg continued increasing during the whole decade both for primary and secondary prevention (Figure S2).

Proportion-wise, the percentage of atorvastatin 80 mg among all atorvastatins dispensed in primary prevention, increased from 0.8% in 2012 to 1.6% in 2018, and decreased to 1.4% in 2021. The percentage of rosuvastatin 40 mg among all rosuvastatins in primary prevention increased from 4.4% in 2012 to 8.2% in 2018, and decreased to 7.6% in 2021. In secondary prevention, percentage of atorvastatin 80 mg among all atorvastatins increased from 6.9% in 2012 to 13.1.% in 2018, and decreased to 11.6% in 2021. The percentage of rosuvastatin 40 mg among all rosuvastatins in secondary prevention increased from 7.5% in 2012 to 16.7% in 2017, and decreased to 11.0% in 2021. 

Both PCSK9 inhibitors (alirocumab and evolocumab) dispensed since 2020 were reimbursed only for secondary prevention. Dispensation of fenofibrate increased modestly from 207.4 to 513.8 thousand units in primary prevention and from 111.8 thousand to 208.2 thousand units in secondary prevention in 2012 and 2021, respectively (Supplementary Figure S3).

## 4. Discussion

To the best of our knowledge, this is the first report on the state-reimbursed LLM dispensation in Latvia. We have demonstrated a steady increase in the numbers of dispensed statin and non-statin LLMs over a decade from 2012 to 2021. The findings reflect the improved implementation of the recommendations by international guidelines and the need for more intensive lipid-lowering as the LDL-cholesterol goals become lower for both primary and secondary ASCVD prevention [2,7]. Our data show that statin usage increased in both CVD prevention levels: it tripled in primary prevention and almost doubled in secondary prevention within a decade. Ezetimibe use in secondary prevention increased nearly 26 times, and in primary prevention almost three times within two years since it became reimbursed for familial hypercholesterolemia.

The trend of substantially more dispensed LLMs in the latter years could not be explained by better reimbursement conditions. The percentage reimbursement according to the ICD codes remained the same, and the proportion of LLMs covered 100% did not change substantially. In fact, cases of full coverage fell by one percentage point throughout the ten years (from 15.6% to 14.6%). The remaining dispensations were covered by either 50% or 75%, and it was only the 50% compensation rate that increased.

There were several noteworthy trends that could be explained by the scientific advances and recommendations in the guidelines. We have witnessed increased use of high-intensity statins both in absolute and relative terms in primary and secondary prevention. In a ten-year period, the proportion of high-intensity doses among all dispensed statins increased from 31.3% in 2012 to 45.2% in 2021. Surprisingly, the increase was particularly prominent in primary prevention (from 17.0% to 34.4%), while in secondary prevention dispensing rates of high-intensity statins have been rather high over the decade (from 39.0 to 54.3%). Importantly, the dispensation of maximum doses of both statins surged: twice for atorvastatin 80 mg and more than 8 times for rosuvastatin 40 mg. In fact, atorvastatin and rosuvastatin were almost exclusively dispensed statins in Latvia (97.0–99.6%) for the whole study period. There was a decrease in 80 mg atorvastatin dispensation mostly due to drop in secondary prevention which could be likely explained by more common use of ezetimibe and rosuvastatin 40 mg. In 2021, the most often dispensed statins were atorvastatin 20 mg, rosuvastatin 20 mg, and rosuvastatin 10 mg, and rosuvastatin was the most commonly dispensed statin in high-intensity doses. 

The dispensation of statins, high-intensity statins, and ezetimibe accelerated starting from 2019 which coincides with the publication of the 2019 ESC/EAS Guidelines for the management of dyslipidaemias that set lower LDL-cholesterol goals, namely, <1.4 mmol/L both in secondary prevention and in primary prevention for very-high risk individuals as opposed to <1.8 mmol/L in 2016 European guidelines in the same populations [2,8]. These 2019 goals were further endorsed by 2021 ESC guidelines on CVD prevention [9].

The most dramatic changes occurred in ezetimibe dispensation, which after being flatlined from 2012 to 2017, rapidly multiplied to 23.5 times in 2021 compared to 2017 or 25.8 times compared to 2012. The results of the IMPROVE-IT trial in 2015, endorsement of the dual combination therapy in the following guidelines, the need for lower LDL-cholesterol levels, and the availability of FDCs of statin (particularly rosuvastatin) with ezetimibe from 2018 obviously accounted for such an increase [10].

In line with the guidelines, we also observed a sharp increase in FDC dispensation of statins with ezetimibe since 2019. Indeed, the role of FDCs in the rise of ezetimibe consumption is further supported by the fact that 90.9% of all ezetimibe dispensations were in the form of FDC with a statin in 2021. Remarkably, every tenth statin was dispensed as an FDC with ezetimibe in 2021: while statin/ezetimibe FDCs accounted for 0.2% of all statins in 2018, this share increased to 10.0% in 2021. It is also noteworthy that of all LLM combinations, the dispensation of high- rather than non-high-intensity statins remained high from 2018 (67.2%) to 2021 (68.3%). 

Some other changes in the availability of lipid-lowering drugs in Latvia can be mentioned. For instance, simvastatin 40 mg became non-available in Latvia after 2015, while rosuvastatin was available only in the years 2014–2019. Concerning simvastatin and fluvastatin, these were rarely prescribed most likely due to their low potency and risk of muscle symptoms for higher doses of simvastatin. Similar data were observed in a national trend study of LLMs for the period of 2010 to 2021 in Lithuania, with very low usage for both [11]. 

Although PCSK9 inhibitors can decrease LDL-cholesterol by around 60% and reduce cardiovascular risk [12,13], in Latvia, due to high cost, they have been reimbursed since July 2020 and only for secondary prevention in specific patients. Their consumption was also modest in Australia [14]. 

Fenofibrate is the only fibrate available in Latvia, and it has been dispensed rarely, mostly in cases of elevated triglycerides. This also goes in line with the guidelines as there is limited evidence of reduced cardiovascular outcomes with fibrates in the era of a more focus on the reduction in LDL-cholesterol and apolipoprotein B-containing lipoproteins.

There is little data in the literature with enough similar studies to make a good comparison with our study. In our view, the contemporary use of statins in Latvia seems similar to national LLM trends in Australia from 2013 to 2019, where atorvastatin and rosuvastatin also were the most prescribed statins (80%). High-intensity statins accounted for 37% of statins in Australia in 2019, which is similar to 41.3% in Latvia in the same year [14]. It should be noted, however, that in our study, we analyzed the total number of units and not prescriptions as in the Australian study.

In a retrospective study by Rodriguez et al. of the period from 2010 to 2018, drug consumption data were analyzed in four countries [15]. It was found that the standardized annual prevalence proportion of statin use increased by 2–3 percentage points in the 8-year period from 2010 until 2018 in all the studied regions: the Region of Southern Denmark (from 21.0% to 22.3%), Udine (Italy) from 12.9% to 14.3%, and in Spain from 20.3% to 23.2%. It should be noted, however, that the methodology and measured estimates were different from our study and cannot be directly compared. In the study by Rodríguez et al., individuals with at least one prescription for the drug each year were counted as users, while we estimated only the numbers of dispensed LLMs and not the individual patient data. 

Marques-Vidal et al. performed a population-based cross-sectional study of adults in Geneva, Switzerland, in which the medications used by individuals to treat dyslipidemia were determined in the period from 2005 to 2019 [16]. This study was based on yearly health examination surveys and data on about 10 000 individuals. The authors found that the use of highly potent statins increased from 50.0% to 87.5% and third-generation statins (defined as rosuvastatin only) from 0% to 47.5% in 2009 and 2015. Interestingly, single-pill combination use was very low, at only 0.6% in 2019. In this study, however, the definition of a high-potency statin differed from the commonly used high-intensity definition. It was defined by the statin name without considering the doses, and apparently, all atorvastatin and rosuvastatin user cases were considered as high-potency statins. In our study, we used the widely accepted 2014 definition of high-intensity statins by the ACC/AHA [7]. 

In our opinion, compared to several other reports, the advantage of our study is that we present ten-year systematic data on the whole country and not only for one region. We also detailed the characterization of types and doses of LLMs and their FDCs; moreover, we took into account the diagnostic codes for prescriptions which allowed us to have an insight into the use of LLMs in primary and secondary prevention.

Our study has several limitations. First, we had no data on unique users of the LLMs; therefore, we do not have an estimate of how many patients are on what medications in Latvia. This will be the focus of our future research. Assessment of LLMs may be further complicated by the changes of therapies and doses within one year for a given patient as well as using various combinations such as a statin plus ezetimibe dispensed separately or an FDCs of lower dose statin and ezetimibe combined with another LLM containing the same statin, a practice that sometimes is observed in Latvia. Second, we are aware that some of the ICD-10 codes defined as primary prevention cases in this study may in fact still be secondary prevention patients. For example, a patient with coronary artery disease may have a prescription for an FDC containing a statin and one or two antihypertensive agents with the diagnosis code for arterial hypertension. Third, we only analyzed the state-reimbursed LLM dispensation because the data on LLMs purchased fully by patients are of limited availability and not shared by the State Agency of Medicines. Based on our experience and personal communications we would estimate that the share of LLMs obtained outside the reimbursement system would be well below 5% of all LLMs consumed. Moreover, drug coverage by private insurers seems negligible in Latvia, especially for LLMs. Thus, in our view, the data presented here are a good estimate of the general trends of LLM consumption in Latvia.

## 5. Conclusions

The dispensing rate of statins doubled, and use of ezetimibe increased more than 25 times in Latvia over a decade. The proportion of high-intensity statins increased from one third to almost half of all statins. Fixed-dose statin combinations with ezetimibe became frequently used.

## Data Availability

The data presented in this study are available on request from the corresponding author.

## Supplementary Materials

The following supporting information can be downloaded at: https://www.mdpi.com/article/10.3390/jcm12196390/s1, Figure S1: Dispensing rates of atorvastatin 80 mg dose in primary and secondary prevention during the decade; Figure S2: Dispensing rates of rosuvastatin 40 mg dose in primary and secondary prevention during the decade; Figure S3: Annual dispensation of fenofibrate for primary and secondary prevention (2012–2021); Table S1: Diagnostic codes attributed to primary and secondary cardiovascular prevention and non-classified cases; Table S2: Numbers of units of lipid-lowering drugs dispensed per year (2012–2021).

## Abbreviations

ACC	American College of Cardiology
AHA	American Heart Association
ASCVD	Atherosclerotic cardiovascular disease
CVD	Cardiovascular disease
FDC	Fixed-dose combination
IBM SPSS	International Business Machines Statistical Package for the Social Sciences
ICD-10	International Classification of Diseases 10th revision
INN	International Nonproprietary Name
LDL-cholesterol	Low-density lipoprotein cholesterol
LLM	Lipid-Lowering Medication
M	Million
mg	Milligram
PCSK9	Proprotein convertase subtilisin/kexin type 9
