# Supplementary material for: National Trends in the Use of State-Reimbursed Lipid-Lowering Medications in Latvia (2012–2021)
Source: J Clin Med. 2023 Oct 6;12(19):6390. doi: 10.3390/jcm12196390 (PMC10573322; doi:10.3390/jcm12196390)
Supplement: Supplementary file 1 [file jcm-12-06390-s001.zip › jcm-2585313-supplementary.pdf]

Supplementary Table S1. Diagnostic codes attributed to primary and secondary cardiovascular prevention and non-classified cases.

Prevention level	ICD-10 code	Diagnosis
**Primary prevention**	B23.8	HIV disease resulting in other specified conditions
	E11.9	Type 2 diabetes mellitus without complications
	E78.0	Pure hypercholesterolaemia
	E78.01*	Familial hypercholesterolemia*
	E78.1	Pure hyperglyceridaemia
	E78.2	Mixed hyperlipidaemia
	I10	Essential (primary) hypertension
	I11.0	Hypertensive heart disease with (congestive) heart failure
	I11.9	Hypertensive heart disease without (congestive) heart failure
	I12.0	Hypertensive renal disease with renal failure
	I12.9	Hypertensive renal disease without renal failure
	I13.0	Hypertensive heart and renal disease with (congestive) heart failure
	I13.1	Hypertensive heart and renal disease with renal failure
	I13.2	Hypertensive heart and renal disease with both (congestive) heart failure and renal failure
	I13.9	Hypertensive heart and renal disease, unspecified

	I15.0	Renovascular hypertension
	I15.1	Hypertension secondary to other renal disorders
	I15.2	Hypertension secondary to endocrine disorders
	I15.8	Other secondary hypertension
	I15.9	Secondary hypertension, unspecified
	Z00.0	General medical examination
	Z94.0	Kidney transplant status
**Secondary prevention**		
	G45.0	Vertebro-basilar artery syndrome
	G45.1	Carotid artery syndrome (hemispheric)
	G45.2	Multiple and bilateral precerebral artery syndromes
	G45.3	Amaurosis fugax
	G45.4	Transient global amnesia
	G45.8	Other transient cerebral ischaemic attacks and related syndromes
	G45.9	Transient cerebral ischaemic attack, unspecified
	I20.0	Unstable angina
	I20.1	Angina pectoris with documented spasm
	I20.8	Other forms of angina pectoris

I20.9	Angina pectoris, unspecified
I21.0	Acute transmural myocardial infarction of anterior wall
I21.1	Acute transmural myocardial infarction of inferior wall
I21.2	Acute transmural myocardial infarction of other sites
I21.3	Acute transmural myocardial infarction of unspecified site
I21.4	Acute subendocardial myocardial infarction
I21.9	Acute myocardial infarction, unspecified
I25.0	Atherosclerotic cardiovascular disease, so described
I25.1	Atherosclerotic heart disease
I25.2	Old myocardial infarction
I25.3	Aneurysm of heart
I25.4	Coronary artery aneurysm and dissection
I25.5	Ischaemic cardiomyopathy
I25.6	Silent myocardial ischaemia
I63.0	Cerebral infarction due to thrombosis of precerebral arteries
I63.1	Cerebral infarction due to embolism of precerebral arteries
I63.2	Cerebral infarction due to unspecified occlusion or stenosis of precerebral arteries
I63.3	Cerebral infarction due to thrombosis of cerebral arteries
I63.4	Cerebral infarction due to embolism of cerebral arteries
I63.5	Cerebral infarction due to unspecified occlusion or stenosis of cerebral arteries

	I63.8	Other cerebral infarction
	I63.9	Cerebral infarction, unspecified
	I65.0	Occlusion and stenosis of vertebral artery
	I65.1	Occlusion and stenosis of basilar artery
	I65.2	Occlusion and stenosis of carotid artery
	I65.3	Occlusion and stenosis of multiple and bilateral precerebral arteries
	I65.8	Occlusion and stenosis of other precerebral artery
	I65.9	Occlusion and stenosis of unspecified precerebral artery
	I66.0	Occlusion and stenosis of middle cerebral artery
	I66.1	Occlusion and stenosis of anterior cerebral artery
	I66.2	Occlusion and stenosis of posterior cerebral artery
	I66.3	Occlusion and stenosis of cerebellar arteries
	I66.4	Occlusion and stenosis of multiple and bilateral cerebral arteries
	I66.8	Occlusion and stenosis of other cerebral artery
	I66.9	Occlusion and stenosis of unspecified cerebral artery
	Z95.1	Presence of aortocoronary bypass graft
	Z95.5	Presence of coronary angioplasty implant and graft
	Z95.8	Presence of other cardiac and vascular implants and grafts
**Unclassified**		
	E20.8	Other hypoparathyroidism

I63.6	Cerebral infarction due to cerebral venous thrombosis, nonpyogenic
J04.0	Acute laryngitis
J04.1	Acute tracheitis
J40	Bronchitis, not specified as acute or chronic
Z95.0	Presence of electronic cardiac devices
Z95.2	Presence of prosthetic heart valve

Source of the codes: https://icd.who.int/browse10/2019/en#/

* E78.01 code is used for Familial hypercholesterolemia in Latvia since 2018.

Supplementary Table S2. Numbers of units of lipid-lowering drugs dispensed per year (2012-2021).

	2012	2013	2014	2015	2016	2017	2018	2019	2020	2021
**Atorvastatin 10 mg**	4,051,260	4,617,577	4,355,941	4,259,066	4,162,504	4,099,050	3,948,592	3,993,685	3,916,748	3,868,542
**Atorvastatin 20 mg**	7,398,165	8,126,001	8,285,274	8,582,983	9,114,487	10,223,100	10,799,020	11,371,429	11,968,872	12,248,902
**Atorvastatin 30 mg**	141,740	463,201	580,156	640,260	769,810	836,653	849,427	833,540	887,343	967,970
**Atorvastatin 40 mg**	3,351,939	3,645,240	3,561,651	3,692,576	3,941,041	4,419,883	4,729,261	4,933,969	5,037,498	5,011,106
**Atorvastatin 60 mg**	23,640	49,830	95,386	126,967	168,530	210,285	219,075	219,980	235,710	235,650
**Atorvastatin 80 mg**	723,260	939,983	1,071,420	1,192,937	1,408,940	1,679,308	1,799,230	1,700,556	1,605,475	1,533,150
**Rosuvastatin 5 mg**			750	11,430	13,740	12,420	4,920	30		
**Rosuvastatin 10 mg**	1,322,407	1,507,828	1,686,629	1,993,052	2,305,830	2,604,967	2,875,769	4,073,403	5,102,477	5,932,219
**Rosuvastatin 15 mg**	51,212	269,081	405,275	428,386	477,738	536,618	495,645	475,302	527,100	592,536
**Rosuvastatin 20 mg**	1,799,954	2,231,463	2,567,187	3,078,280	3,476,460	3,996,382	4,564,204	6,164,754	8,383,644	10,583,892
**Rosuvastatin 30 mg**	37,534	112,623	171,822	210,248	259,042	290,535	286,311	294,201	340,298	416,013
**Rosuvastatin 40 mg**	226,681	437,716	591,289	734,790	897,932	1,157,455	1,299,212	1,433,369	1,659,781	1,869,988
**Ezetimibe 10 mg**	184,744	179,652	155,862	136,360	151,844	202,594	609,270	1,945,060	3,414,675	4,767,918
**Simvastatin 10 mg**	40,296	616								
**Simvastatin 20 mg**	492,111	473,999	381,892	344,730	316,410	274,810	235,880	212,000	199,800	181,160
**Simvastatin 40 mg**	22,470	15,848	7,000							
**Fluvastatin 80 mg**	38,304	34,776	33,040	30,296	25,876	23,156	22,764	20,076	9,436	9,800
**Alirocumab 75 mg**									10	76
**Alirocumab 150 mg**									38	392
**Evolocumab 140 mg**									114	364
**Fenofibrate 200 mg**	319,185	433,035	486,202	538,209	581,190	641,535	669,360	664,458	698,445	722,055

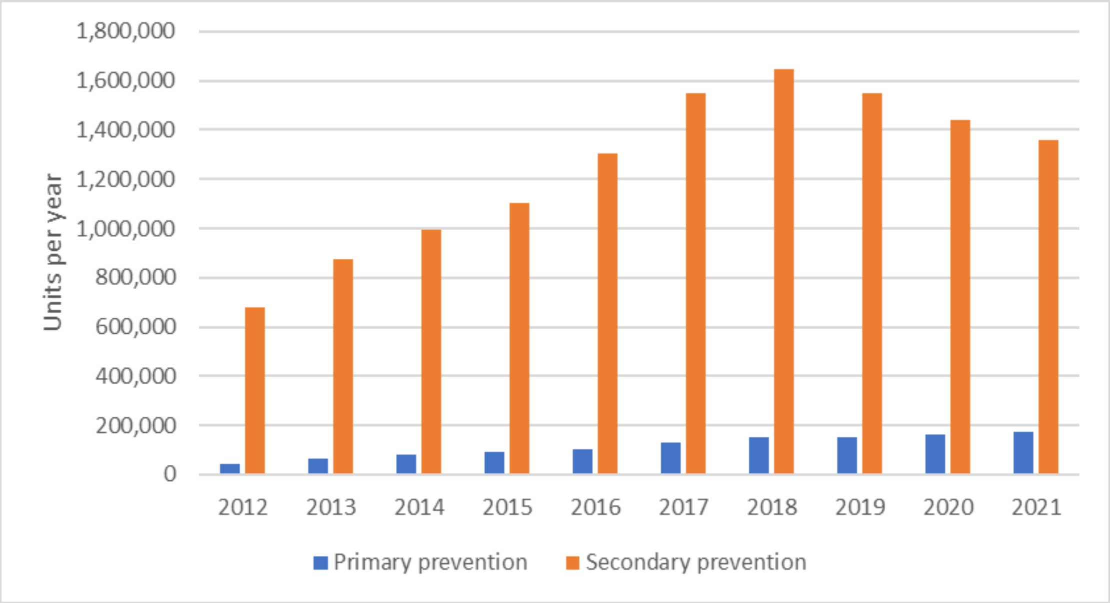

Figure S1. Dispensing rates of atorvastatin 80 mg dose in primary and secondary prevention over a decade.

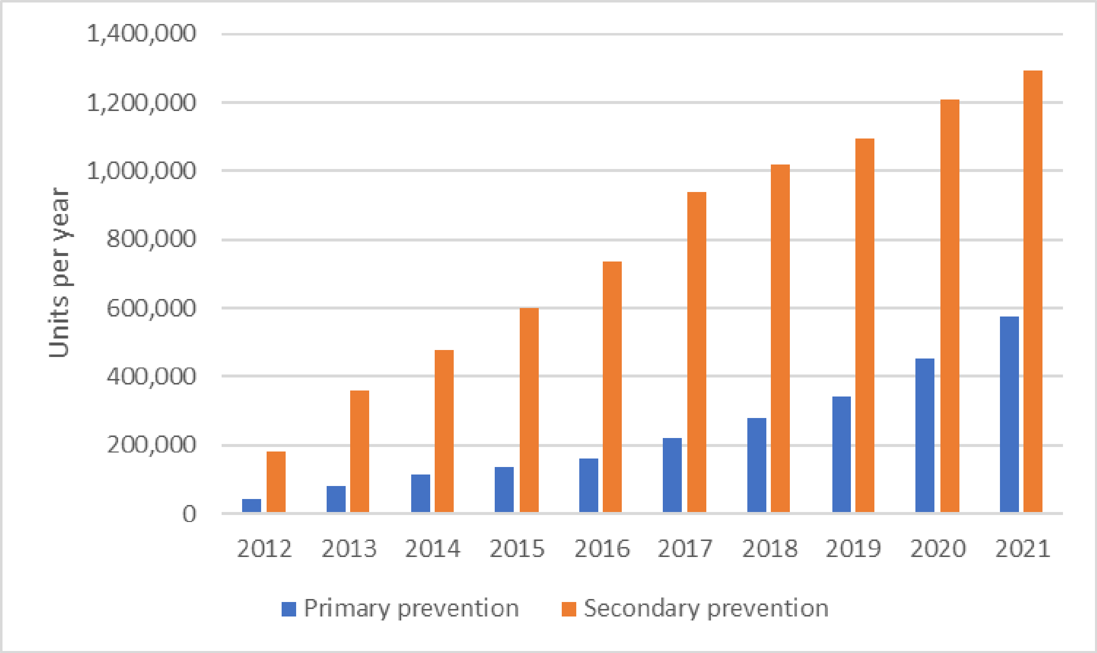

Figure S2. Dispensing rates of rosuvastatin 40 mg dose in primary and secondary prevention over a decade.

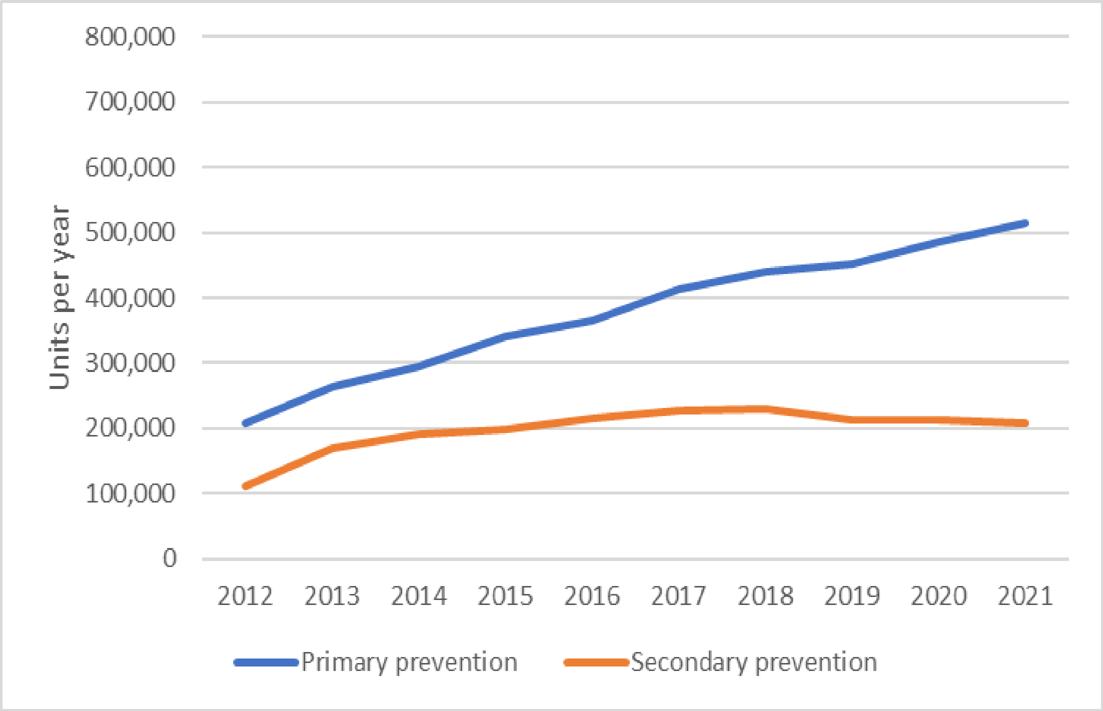

Figure S3. Annual dispensation of fenofibrate for primary and secondary prevention (2012–2021).
